# Development and Validation of a Prognostic Gene Signature Correlated With M2 Macrophage Infiltration in Esophageal Squamous Cell Carcinoma

**DOI:** 10.3389/fonc.2021.769727

**Published:** 2021-12-03

**Authors:** Jiannan Yao, Ling Duan, Xuying Huang, Jian Liu, Xiaona Fan, Zeru Xiao, Rui Yan, Heshu Liu, Guangyu An, Bin Hu, Yang Ge

**Affiliations:** ^1^ Department of Oncology, Beijing Chao-Yang Hospital, Capital Medical University, Beijing, China; ^2^ Medical Research Center, Beijing Chao-Yang Hospital, Capital Medical University, Beijing, China; ^3^ Department of Thoracic Surgery, Beijing Chao-Yang Hospital, Capital Medical University, Beijing, China

**Keywords:** Esophageal squamous cell carcinoma, tumor microenvironment, prognostic biomarker, immunotherapy, M2 macrophage

## Abstract

**Background:**

Esophageal squamous cell carcinoma (ESCC) is the most common type of esophageal cancer and the seventh most prevalent cause of cancer-related death worldwide. Tumor microenvironment (TME) has been confirmed to play an crucial role in ESCC progression, prognosis, and the response to immunotherapy. There is a need for predictive biomarkers of TME-related processes to better prognosticate ESCC outcomes.

**Aim:**

To identify a novel gene signature linked with the TME to predict the prognosis of ESCC.

**Methods:**

We calculated the immune/stromal scores of 95 ESCC samples from The Cancer Genome Atlas (TCGA) using the ESTIMATE algorithm, and identified differentially expressed genes (DEGs) between high and low immune/stromal score patients. The key prognostic genes were further analyzed by the intersection of protein–protein interaction (PPI) networks and univariate Cox regression analysis. Finally, a risk score model was constructed using multivariate Cox regression analysis. We evaluated the associations between the risk score model and immune infiltration *via* the CIBERSORT algorithm. Moreover, we validated the signature using the Gene Expression Omnibus (GEO) database. Within the ten gene signature, five rarely reported genes were further validated with quantitative real time polymerase chain reaction (qRT-PCR) using an ESCC tissue cDNA microarray.

**Results:**

A total of 133 up-regulated genes were identified as DEGs. Ten prognostic genes were selected based on intersection analysis of univariate COX regression analysis and PPI, and consisted of C1QA, C1QB, C1QC, CD86, C3AR1, CSF1R, ITGB2, LCP2, SPI1, and TYROBP (HR>1, p<0.05). The expression of 9 of these genes in the tumor samples were significantly higher compared to matched adjacent normal tissue based on the GEO database (p<0.05). Next, we assessed the ability of the ten-gene signature to predict the overall survival of ESCC patients, and found that the high-risk group had significantly poorer outcomes compared to the low-risk group using univariate and multivariate analyses in the TCGA and GEO cohorts (HR=2.104, 95% confidence interval:1.343-3.295, p=0.001; HR=1.6915, 95% confidence interval:1.053-2.717, p=0.0297). Additionally, receiver operating characteristic (ROC) curve analysis demonstrated a relatively sensitive and specific profile for the signature (1-, 2-, 3-year AUC=0.672, 0.854, 0.81). To identify the basis for these differences in the TME, we performed correlation analyses and found a significant positive correlation with M1 and M2 macrophages and CD8+ T cells, as well as a strong correlation to M2 macrophage surface markers. A nomogram based on the risk score and select clinicopathologic characteristics was constructed to predict overall survival of ESCC patients. For validation, qRT-PCR of an ESCC patient cDNA microarray was performed, and demonstrated that C1QA, C3AR1, LCP2, SPI1, and TYROBP were up-regulated in tumor samples and predict poor prognosis.

**Conclusion:**

This study established and validated a novel 10-gene signature linked with M2 macrophages and poor prognosis in ESCC patients. Importantly, we identified C1QA, C3AR1, LCP2, SPI1, and TYROBP as novel M2 macrophage-correlated survival biomarkers. These findings may identify potential targets for therapy in ESCC patients.

## Introduction

Esophageal cancer (EC) is the seventh leading cause of cancer-related death worldwide due to its high malignancy and poor prognosis, with an estimated 5-year survival rate of approximately 10-15% (1–3). It is estimated that approximately 572034 new cases of esophageal cancer and 508585 deaths due to EC in 2018 worldwide (4). Esophageal Squamous cell carcinoma (ESCC) is the predominant histology of EC, constituting 90% of cases worldwide, and approximately half of the world’s 500,000 new cases occur in China each year (5). More than half of ESCC patients are at an advanced stage when diagnosed (6). Despite recent advances in multidisciplinary therapeutic approaches, its prognosis remains unfavorable due to the high rates of recurrence, metastasis and the resistance to systematic therapy (7). Immunotherapy is a revolutionary treatment approach which has led to marked therapeutic responses among advanced melanoma, non–small cell lung cancer and renal cell carcinoma. There is an increasing interest in the potential of immunotherapy against ESCC to improve the prognosis of patients. Currently, a variety of clinical trials are ongoing to evaluate immunotherapy as a first line treatment for ESCC. However, the evidence to date suggests that only a minority of patients can benefit from it. Therefore, an urgent need remains to identify innovative biomarkers to accurately predict the prognosis of ESCC patients receiving immunotherapy.

The tumor microenvironment (TME) is the environment in which tumor cells live, and is comprised of innate immune cells, including macrophages, dendritic cells, neutrophils, natural killer (NK) cells, myeloid derived suppressor cells (MDSCs), T and B cells, and stromal cells including fibroblasts, endothelial cells and extracellular matrix (ECM) (8). Studies have revealed that the TME is heterogeneous, and that various tumor-infiltrating immune cells play a pro- or anti-tumorigenic role within it (9, 10). It is thought to contribute to inhibiting apoptosis, enabling immune evasion, and promoting proliferation, angiogenesis, invasion and metastasis (11). Notably, the TME is a key target for immunotherapy in cancer patients (12, 13). Tumors with high CD8+ T cell infiltration (“hot” tumors) show the best response to immune checkpoint inhibitors (ICIs). In contrast, patients with “cold” tumors—also called immune deserts, do not benefit from ICIs due to lack of infiltration with CD8+ T cells (14). A previous study reported that several immune-suppressive cell populations were enriched in TME of ESCC, including regulatory T cell (Tregs), exhausted CD8^+^ T, CD4^+^ T and NK cells, M2 macrophages (15). Moreover, the population densities of NK cells and macrophages has been found to significantly related with postoperative prognosis for stage II-III esophageal cancer patients (16). Further research suggested that Tregs infiltration had an association with the pathological response and showed a potential value in predicting cancer-specific survival (17). Macrophages have been confirmed to impact angiogenesis, tumor cell migration, and invasion and are expected to be attractive targets for cancer immunotherapy. Within the TME, macrophages may polarize into anti-tumorigenic M1 or pro-tumorigenic M2 phenotypes (7, 11). Tumor-associated macrophages (TAM) can promote genetic stability, nurture cancer stem cells, and contribute to tumor progression and metastasis. TAM infiltration is associated with poor responses to chemotherapy and overall poor prognosis (2). Yamamoto et al. confirmed that pre–therapeutic M2 macrophage infiltration would be a useful biomarker for predicting the response to neoadjuvant chemotherapy (NAC) compared with other immune cells in EC patients (18). Moreover, it has been reported that FOXO1 upregulation in tumor tissues drive the polarization of M0 macrophages and infiltration of M2 macrophages into the TME, resulting in worse prognosis in ESCC patients (19). More recently, CSF-1/CSF-1R blockade has gained widespread attention as a TAM-targeted treatment in cancer research (20, 21). Overall, a better understanding of the status of TME in ESCC patient tumors can help to characterize their immunogenomic profile and improve outcomes.

ESTIMATE (Estimation of STromal and Immune cells in MAlignant Tumor tissues using Expression data) is an algorithm designed to analyze cell purity by calculating the ratio of immune and stromal components based on gene expression (22). In our current study, we utilized ESTIMATE and the CIBERSORT algorithm to quantify the level of tumor immune infiltration of 95 ESCC samples from the TCGA database and identified a predictive 10-gene signature associated with poor prognosis of patients. We further verified its prognostic value in the GEO dataset and confirmed its independent prognostic effect. This work, for the first time, establishes a novel M2 macrophage-related gene signature in ESCC and may be used to predict patient outcomes. Moreover, we validated five of the ten genes (C1QA, C3AR1, LCP2, SPI1 and TYROBP) as independently associated with poor survival and tightly related with macrophage M2 surface biomarkers by qPCR, which may provide new therapeutic avenue for ESCC.

## Materials and Methods

### Data Download and Preparation

The level 3 gene expression profile and corresponding clinical information of ESCC patients were downloaded from UCSC Xena (dataset ID: TCGA-ESCA-sampleMap/HiSeqV2, https://xenabrowser.net/datapages/). The gene expression profile was measured experimentally using the Illumina HiSeq 2000 RNA Sequencing platform by the University of North Carolina TCGA genome characterization center. Gene expression was provided as gene-level transcription estimates with units as log2(x+1) transformed RSEM normalized count. Low-expression genes with mean expression values below 1 RSEM in all samples were filtered out using the “limma” package in R version 4.0.2 software. The original data included 185 tumor tissues and 11 adjacent tissues. Among these, 96 samples were histologically diagnosed as squamous cell carcinoma, including 1 paired metastasis tissue. The clinical information of the patients is shown in **Supplemental Table 1**. The risk score model was further validated using the GSE53624 dataset from the Gene Expression Omnibus (GEO, http://www.ncbi. nlm.nih.gov/geo/). The screening process of the validation dataset is provided in **Supplemental Figure 1**. The GSE53624 dataset included 119 paired tumor and normal ESCC tissues based on GPL18109 platform (Agilent-038314 CBC Homo sapiens lncRNA + mRNA microarray V2.0).

### Estimation of Stromal and Immune Components of TME

Immune, stromal, and ESTIMATE scores of the samples were calculated using the estimate R package. We determined the optimal cutpoint based on the function “surv_cutpoint” from the survminer R package. Kaplan–Meier analyses were performed using the survival and survminer packages in R to illustrate the correlation of immune/stromal scores and patient overall survival (OS). The log-rank test was applied to verify the results.

### Identification of Differentially Expressed Genes (DEGs)

According the optimal cutpoint, immune and stromal scores were divided into high/low groups, respectively. DEGs were identified using the R package, limma. The threshold set for up- and down-regulated genes was a |log 2 foldchange (FC)| > 1 and false discovery rate (FDR) < 0.05. Heatmaps were plotted with the package pheatmap.

### Enrichment Analyses and Protein-Protein Interaction (PPI) Network

Functional enrichment analyses of the DEGs with the Kyoto Encyclopedia of Genes and Genomes (KEGG) and gene ontology (GO) were performed with the R package “clusterProfiler”, “org.Hs.eg.db”, “enrichplot” and “ggplot2”. GO enrichment includes biological processes (BP), cellular component (CC) and molecular function (MF). Categories with a p- and q-value of <0.05 were considered significantly enriched. All of the DEGs were uploaded into the STRING (https://string-db.org/) database (v 11.0) to obtain PPI networks, with a combined score > 0.4 considered statistically significant. Cytoscape (version 3.7.1) was used to reconstruct the network. Network nodes represent proteins and edges represent protein-protein associations.

### Construction and Validation of 10-Gene Risk Score Model

Univariate cox regression analysis was performed to examine the prognostic value among ESCC patients. 36 genes with p < 0.05 were identified as prognostic DEGs and were visualized using the forest diagram. Multivariate analyses were performed to develop the 10-gene risk score model. The model was based on expression data multiplied by Cox regression coefficients. The final risk score model formula was as followed: Risk score = [Expression level of C1QA * (0.32596)] +[Expression level of C1QB * (1.40234)]+[Expression level of C1QC* (-1.36687)+ [Expression level of CD86 * (0.35249)]+[Expression level of C3AR1* (-0.07155)+ [Expression level of CSF1R * (0.28413)]+[Expression level of ITGB2 * (-0.52918)]+[Expression level of LCP2* (-0.22934)+ [Expression level of SPI1 * (1.05328)]+[Expression level of TYROBP* (-0.87910)]. Patients were divided into low-risk and high-risk groups according to the optimal cutpoint. The K-M survival curves for the groups with low or high risk were performed. The predictive ability of the model was assessed by the survival receiver operating characteristic (ROC) package in R software and was used to compare the area under the curve (AUC) of our gene signature and those derived in two other published studies. To confirm the risk model’s independent prognostic value, univariate and multivariate Cox survival analyses were performed with select clinical factors. Finally, external data from GSE53624 was applied to verify the reliability of the gene signature’s impact on the prognosis of the patients. The differential expression analysis was performed based on 119 paired tumor and normal samples.

### Estimation of Immune Infiltration

CIBERSORT in combination with the LM22 method was carried out to quantify the abundances of immune cell types in the TME. The 22 types of infiltrating immune cells inferred by CIBERSORT include B cells, T cells, natural killer cells, macrophages, dendritic cells, eosinophils and neutrophils. The CIBERSORT p-value reflects the statistical significance of the results, only tumor samples with p<0.05 were used for further analysis.

### Construction and Validation of the Nomogram

A nomogram was established based on the risk score and select clinicopathologic characteristics including age, gender and stage to predict the survival probability of 1-, 2-, and 3-year OS of ESCC patients. The nomogram and calibration plots were generated based on the rms R package. The calibration curve of the nomogram was plotted to evaluate the prediction possibilities against the observed rates.

### Gene Set Enrichment Analysis (GSEA)

GSEA was carried out to evaluate associations between immune pathways and 10-gene signature using the software GSEA-4.1.0. Hallmark (h.all.v7.4.symbols.gmt) and C7 gene sets (c7.all.v7.4.symbols.gmt, Immunologic Signatures) were downloaded from Molecular Signatures Database (MSigDB) as the target sets. Only gene sets with NOM p < 0.05 and FDR q < 0.25 were considered significant.

### cDNA Microarray Chip and Real-Time PCR

Tissue cDNA chips including cDNA from 67 cases of esophageal squamous cell carcinoma tissue and 28 peri-carcinoma tissues with complete clinical and survival information were purchased. A cDNA microarray chip (cDNA-HEsoS095Su01,Outdo Biotech Company, Beijing, China) was used for the tumor or peritumor tissue samples in this study. The mRNA expression levels of hub genes and immune cell surface biomarkers were detected by Hieff qPCR SYBR Green Master Mix (Low Rox Plus) (YEASEN Biotech Co., Ltd). The qPCR protocol was 95°C for 5min, 40 cycles at 95°C for 10 s, and 60°C for 60 s. Primers used in this study are presented in **Supplemental Table 2**. The relative expression levels of hub genes were determined by the 2^−ΔΔCT^ method.

### Statistical Analysis

The correlation analysis was performed using the Spearman method. Survival curves were compared using the Kaplan-Meier method and the log-rank test. R version 4.0.2 and GraphPad 5.0 were used to perform statistical analysis. The results are presented as mean ± standard deviation (mean ± SD). Differences between groups were evaluated by the Wilcoxon rank-sum test. All tests were two-sided and p < 0.05 indicates statistical significance.

## Results

### Immune and Stromal Scores Are Significantly Correlated With ESCC Survival

**Figure 1:**
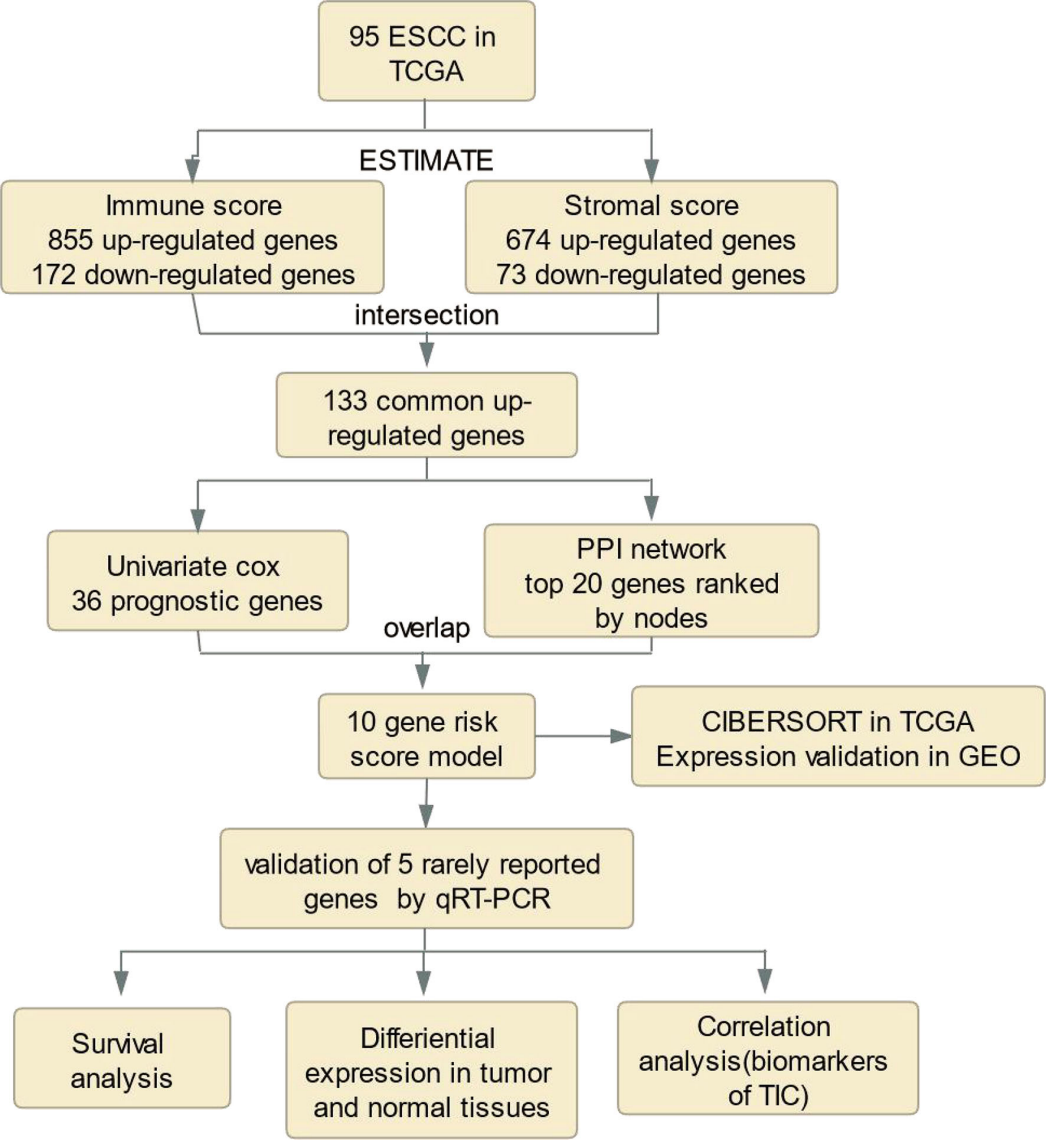
Analysis workflow of this study.

**Figure 2:**
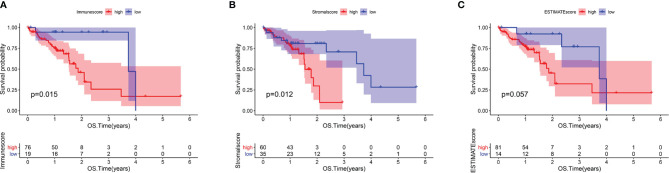
Immune and Stromal scores were correlated with overall survival of ESCC. **(A)** Kaplan–Meier survival analysis for ESCC patients grouped into high or low score in Immunescore determined by the optimal cutoff. p = 0.015 by log-rank test. **(B)** Kaplan–Meier survival curve for Stromalscore with p = 0.012 by log-rank test. **(C)** Survival analysis with Kaplan–Meier method for ESCC patients grouped by ESTIMATEScore (p = 0.057 by log-rank test).

The analysis process for this study is presented in **Figure 1**. We estimated the immune/stromal/ESTIMATE scores of 95 ESCC tumors using the ESTIMATE algorithm. The immune scores varied from -1389.05 to 3395.19, the stromal scores ranged from -1859.96 to 1301.57, the ESTIMATE scores ranged from -2672.1 to 3970.4. All samples were categorized to high/low groups with the optimal cutpoint. Using Kaplan-Meier analysis, we found that patients with higher immune scores experienced poorer overall survival (OS) compared to those with low scores (p=0.015, **Figure 2A**). In agreement, stromal scores were inversely correlated with OS (p=0.012, **Figure 2B**). The high ESTIMATE score group also showed poorer OS in comparison to the low score group (p=0.057, **Figure 2C**). These findings demonstrate that the immune/stromal components in TME are significant in predicting the prognosis of ESCC patients. We further explored the potential relationship between the clinicopathological characteristics and the immune/stromal scores, but found no significant associations.

### Differential Expression and Functional Enrichment Analysis

**Figure 3:**
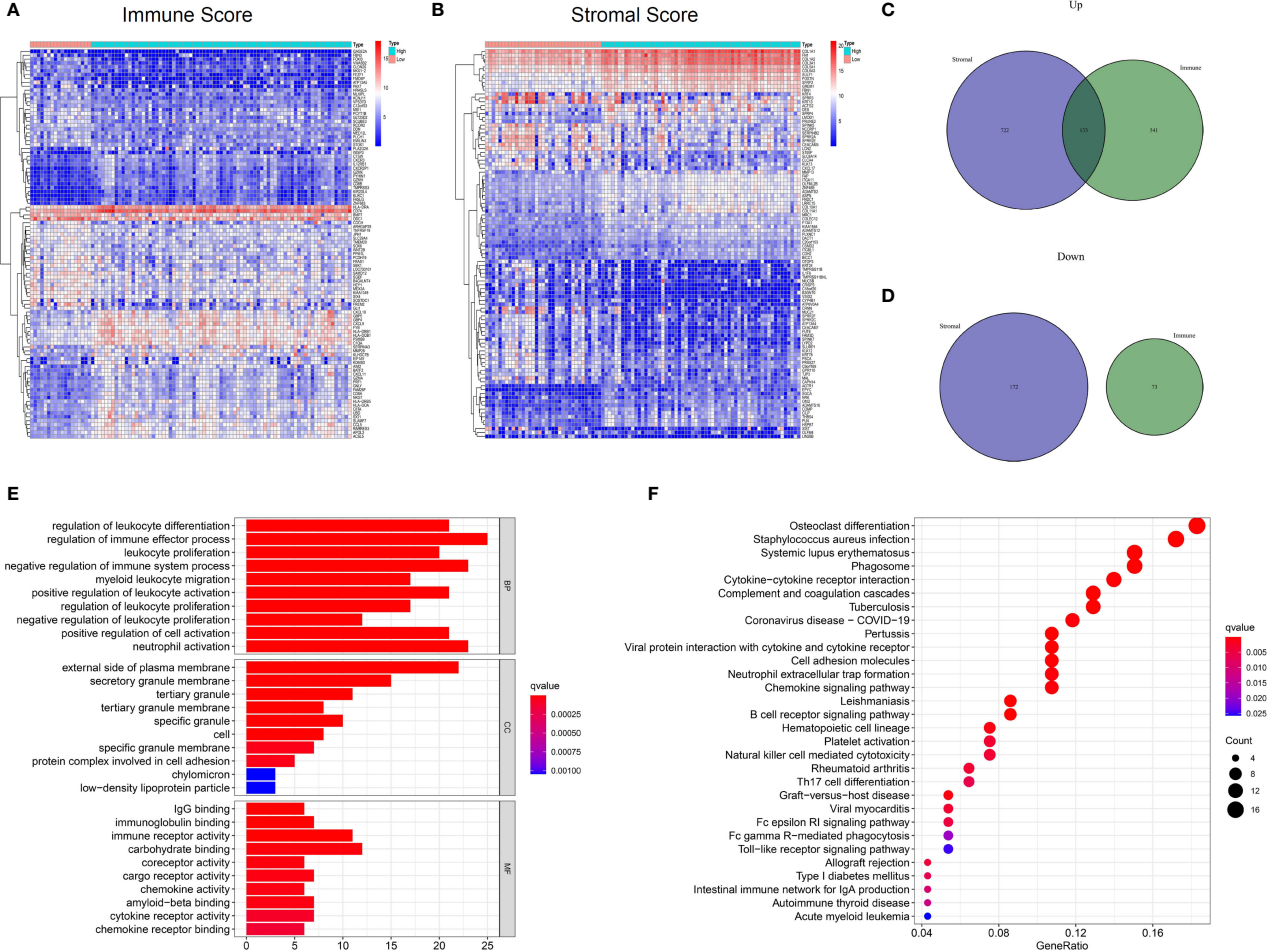
Differential expressed genes identification and enrichment analysis of GO and KEGG. **(A)** Heatmap for DEGs between high and low Immuneacore groups. Row name of heatmap is the gene name, and column name is the ID of samples which not shown in plot. **(B)** Heatmap for DEGs in Stromalscore, similar with **(A)**. **(C, D)** Venn plots for common up-regulated and down-regulated DEGs shared by Immunescore and Stromalscore. **(E)** The top ten biological processes (BP) cellular components (CC) and molecular functions (MF) for GO analysis, respectively. **(F)**The top 30 KEGG enrichment signaling pathways for 133 DEGs, terms with p and q < 0.05 were determined to be enriched significantly.

To investigate the potential relationship between gene expression profiles and immune/stromal scores, we performed differential expression analysis of high- and low-score groups. According to the analysis (high immune score vs low immune score), a total of 747 DEGs were selected, which contained 674 up-regulated and 73 down-regulated DEGs. Similarly, 1027 DEGs were obtained based on differential analysis of stromal scores, consisting of 855 up-regulated and 172 down-regulated DEGs. Hierarchical clustering (**Figures 3A, B**) showed that DEGs were significantly dysregulated between the two groups. Furthermore, after intersection of the two lists of genes (**Figures 3C, D**), we obtained 133 up-regulated DEGs shared by immune and stromal groups. These DEGs can be regarded as candidate TME-related genes.

Furthermore, functional enrichment analysis was performed to find the potential mechanism of 133 DEGs in the TME. The top 10 significant results of the enrichment analysis for BP, CC and MF are displayed in **Figure 3E**. For BP, DEGs were mainly enriched in immune effector processes, negative regulation of immune system processes, and positive regulation of cell activation. In the CC group, DEGs were mainly enriched in the external side of plasma membrane, protein complex involved in cell adhesion, and secretory granule membrane. In the MF classification, the top terms included immunoglobulin binding, immune receptor activity, chemokine activity, cytokine receptor activity, and chemokine receptor binding. For the KEGG analysis (**Figure 3F**), DEGs were mainly enriched in the Natural killer cell mediated cytotoxicity, Toll−like receptor signaling pathway, viral protein interaction with cytokine and cytokine receptor, cell adhesion molecules, Th17 cell differentiation, and chemokine signaling pathways. Collectively, these findings suggest that the functions of these genes are immune-related.

### Intersection of PPI Network and Univariate Cox Regression Analysis

**Figure 4:**
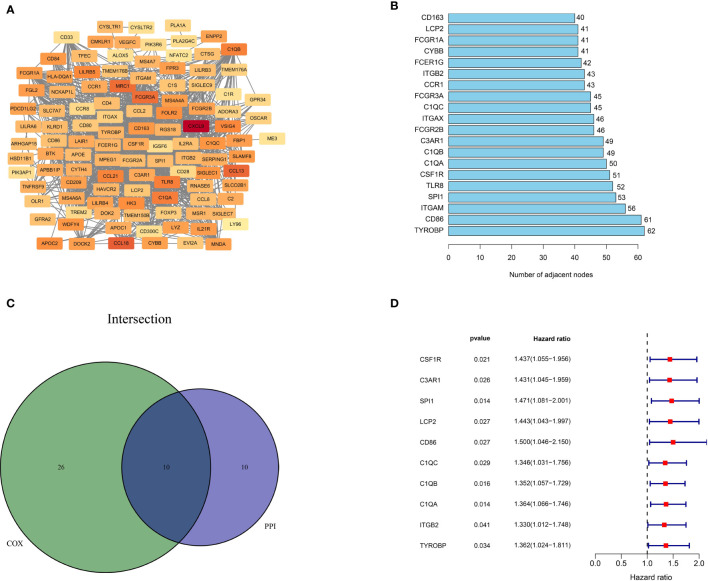
Protein-protein interaction network and univariate COX analysis. **(A)** PPI network constructed with the nodes with interaction confidence value > 0.4. **(B)** The top 20 genes according to the number of nodes. **(C)** Venn plot for 10 hub prognostic DEGs. **(D)** The 10 hub prognostic genes with p < 0.005 in univariate COX regression analysis.

To better illustrate the interrelationship among these DEGs, we used the STRING database and the Cytoscape software to construct a PPI network. As depicted in **Figure 4A**, the color from light to dark represents the ascending logFC value. The barplot (**Figure 4B**) shows the top 20 hub genes ranked by the number of nodes. Next, we carried out univariate cox regression analysis and identified 36 genes significantly associated with the poor OS of ESCC patients (HR>1, p<0.05). Finally, the 10 independent prognostic genes in the PPI and univariate cox regression analysis were overlapped to identify the common hub genes, including C1QA, C1QB, C1QC, CD86, C3AR1, CSF1R, ITGB2, LCP2, SPI1 and TYROBP (**Figure 4C**). The forest diagram (**Figure 4D**) illustrates the relationships between these 10 genes and prognosis. The Kaplan-Meier curve confirmed that high expression of 9 of the genes (C1QA, C1QB, CSF1R, C3AR1, ITGB2, LCP2, SPI1, TYROBP) were significantly related to poor OS (**Supplemental Figures 2A–I**, p<0.05). Moreover, for those variables in which survival curves intersected, we carried out landmark analysis discriminating between events occurring before and after 3 years of follow-up to eliminate immortal time bias, which confirmed that all genes are significantly related to overall survival before 3 years (**Supplemental Figure 3B**, p-value<0.05).

### Correlations Between Immune Infiltration and Prognostic Genes

**Figure 5:**
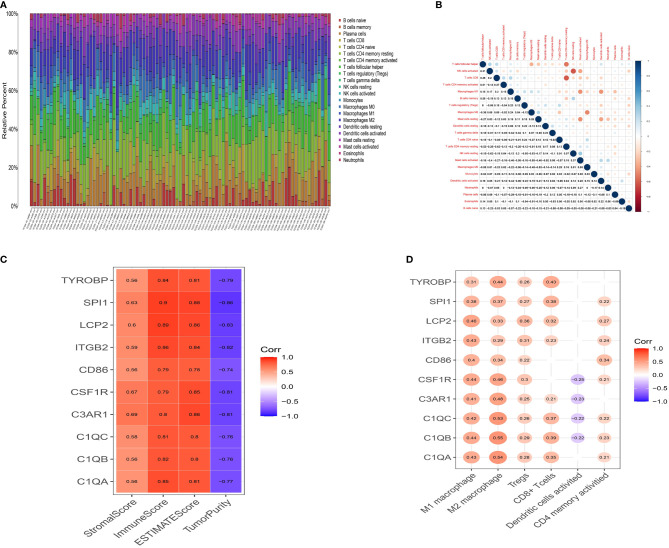
Landscape of tumor infiltration cells in ESCC. **(A)** Barplot for the proportion of 22 types of immune cells in ESCC tissues. Column names are sample ID. **(B)** Heatmap for the correlations between the levels of immune cells among ESCC samples. **(C)** Heatmap for correlation between 10 genes and StromalScore, ImmuneScore, ESTIMATEScore and TumorPurity (p<0.05). **(D)** Heatmap for correlation between 10 genes and six immune cells. The blank means that the p-value is nonsignificant.

The CIBERSORT algorithm was then used to quantify the proportion of tumor-infiltrating immune subsets and further understand the correlation of hub genes with the immune TME. **Figure 5A** shows the relative proportions of immune cells in each ESCC sample. Positive and negative correlations between immune cells were obtained, as displayed in **Figure 5B**. There was a moderate correlation between macrophage M0 and macrophage M2 (r=-0.49) in ESCC. CD8 T cells were moderately and positively correlated with activated CD4 memory T cells (r=0.27), while negatively correlated with resting CD4 memory T cells (r=-0.62). M1 macrophages were positively associated with Tregs (r=0.23) and M2 macrophages (r=0.24), and negatively associated with activated dendritic cells (r=-0.44). These results reveal that different kinds of immune cells interfere with each other in TME. Pearson correlation analysis was further performed to compare the expression of ten genes and the results of ESTIMATE. All ten genes were strongly positively correlated with ImmuneScore but negatively correlated with TumorPurity (**Figure 5C**, p <0.05), demonstrating lower the purity of the tumor and increased immune cells in the TME. Moreover, we searched the CCLE (Cancer Cell Line Encyclopedia) database to help clarify whether these genes are overexpressed in immune cells or tumor cells. The expression values for the ten genes and classic epithelial markers in esophageal cancer cell lines was downloaded. All ten genes showed extremely low expression in esophageal cancer cell lines (**Supplemental Figure 4**), with average expression values (RPKM) of: C1QA 0.0005, C1QB 0.0002, C1QC 0, C3AR1 0.0385, CD86 0.2466, CSF1R 0.1609, ITGB2 1.0324, LCP2 0.0123, SPI1 0.0255, TYROBP 0.0086. Comparably, the epithelial markers selected as controlled genes showed relatively high expression CDH1 57.414, CLDN4 60.703, CLDN7 50.385, MUC1 13.366, TJP3 4.5215. Therefore, these combined evidences lead us to believe that these genes are likely to be specifically expressed in immune cells. To investigate this further, we performed a single-gene immune infiltration analysis for each gene to illustrate its relationships with various immune cells. Most of the genes were related to certain types of immune cells. As depicted in **Figure 5D**, three kinds of tumor infiltrating immune cells (TICs) were positively correlated with all genes, including M1 macrophages, M2 macrophages, and regulatory T cells (Tregs). M2 macrophages had a higher correlation with C1QA (r=0.54), C1QB (r=0.55), C1QC (r=0.53), C3AR1 (r=0.48), CSF1R (r=0.46) and TYROBP (r=0.44). M1 macrophages were moderately linked with LCP2 (r=0.46), ITGB2 (r=0.43), CSF1R (r=0.44), C1QA (r=0.43), C1QB (r=0.44), C1QC (r=0.42) and C3AR1 (r=0.41). All genes were positively associated with M2 macrophages (**Supplemental Figures 5 A–J**, p<0.05). These findings further confirm that these prognostic genes are related to the immune activity of the TME, especially M2 macrophages.

### Construction of a Risk Score Model and Validation of Its Predictive Value

**Figure 6:**
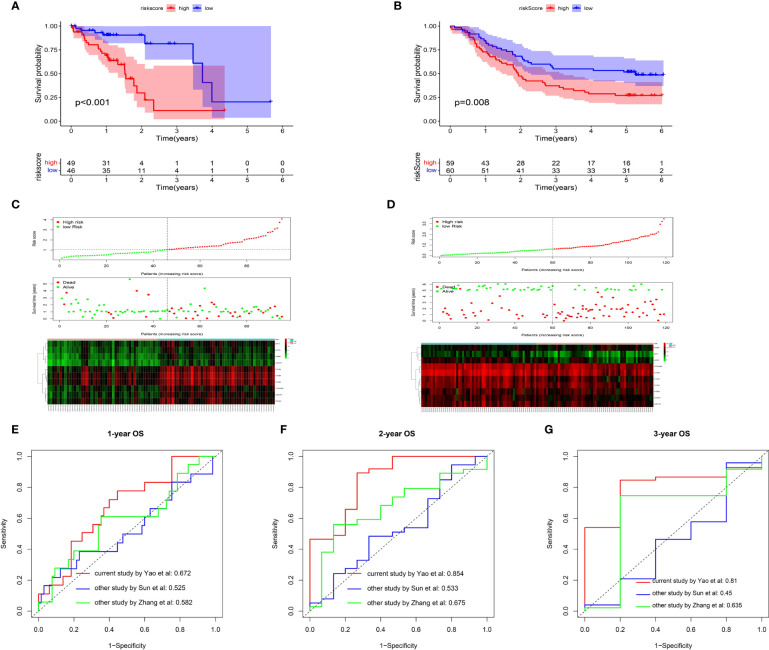
Prognostic analysis of the risk score model. **(A, B)** Kaplan-Meier survival curve of risk score model among ESCC patients in TCGA cohort **(A)** and GEO cohort **(B)**.The high-risk group show the poorly prognosis (p<0.05). **(C, D)** Relationship between the risk score (upper) and the expression of ten prognostic genes (lower) in TCGA cohort **(C)** and GEO cohort **(D)**. **(E, F)** Time-dependent ROC analysis was performed to compare the three models in predicting 1-year **(E)**, 2-year **(F)** and 3-year **(G)** OS.

Utilizing the multivariate Cox regression analysis, we established a 10-gene risk score model. The risk model met the proportional hazard assumption based on the Schoenfeld Individual Test results which showed that each covariate is not statistically significant (**Supplemental Figure 2A**, p>0.05). We then calculated the risk score for each sample and divided patients into high- or low-risk group according to the optimal cutpoint by maximally selected rank statistics. Kaplan-Meier analysis suggested that patients in the high-risk group have significantly shorter OS than those in the low-risk group (**Figure 6A**, p<0.001). The distribution of risk scores, survival status, and ten-gene expression levels among patients in the high- and low-risk group are given in **Figure 6C**. To evaluate the independent predictive value of our risk model, univariate and multivariate analyses were performed. The results showed gender, stage, N-stage and risk score were significantly associated with OS in univariate analysis (p<0.05). In the multivariate analysis, only risk score was associated with OS (HR=2.104, 95% CI=1.343-3.295; p=0.001) (**Table 1**). To verify the prognostic value and reliability of our results, the risk score model was further validated using the GEO dataset, which includes 119 ESCC patients. All patients were divided into high- and low-risk group according to the previous formula. In agreement with the training cohort, patients in the high-risk group had significantly worse OS than the low-risk group (**Figure 6B**, p-value=0.008). The univariate and multivariate analyses of risk score and other clinical characteristics confirmed that the risk score model was an independent prognostic indicator (HR=1.6915, 95% CI:1.053-2.717; p=0.0297, **Table 2**). We also carried out differential expression analysis of the complete ten-gene signature in the validation dataset, The Wilcoxon rank sum test revealed that the expression of 9 genes (C1QA, C1QB, C1QC, C3AR1, ITGB2, LCP2, SPI1, TYROBP) in the tumor samples were significantly higher than that in matched normal adjacent tissue (**Supplemental Figures 6A–J**, p<0.05). Time-dependent ROC curve analysis demonstrated that during 1-, 2-, and 3-year follow-up, the area under the curve (AUC) values were 0.672, 0.854, and 0.81 respectively (**Figures 6E–G**) Recently, Sun et al. identified a prognostic gene signature among patients with ESCC (23). Zhang et al. constructed a prognostic model based on immune-related genes to predict prognosis of esophageal cancer (24). We calculated C-indexes to compare the prognostic values of our model and theirs. As shown in **Figures 6E–G**, the concordance index of the risk score model for the 1-, 2-, and 3-year OS was higher than the other two studies, indicating that our risk score model may have better performance in predicting prognosis. Taken together, the results confirm that the risk score model is an reliable and independent prognostic factor for ESCC patients.

**Table 1:** Univariate and Multivariate cox regression analysis-TCGA cohort.

Variables	Univariate analysis		Multivariate analysis	
	HR (95%CI)	P value	HR (95%CI)	P value
Risk score	2.113 (1.388-3.215)	<0.001	2.104 (1.343-3.295)	0.001
Stage (III+IV/I+II)	2.390 (1.165-4.903)	0.017	1.632 (0.771-3.453)	0.200
Grade (2 + 3/1)	1.616 (0.557-4.684)	0.377		
pT (3 + 4/1+2)	1.279 (0.615-2.662)	0.510		
pN (1 + 2+3/0)	1.988 (0.962-4.108)	0.063		
pM (1/0)	2.265 (0.671-7.644)	0.188		
Gender (Male/Female)	5.266 (1.221-22.71)	0.026	3.277 (0.745-14.405)	0.116
Age (≥65/65)	1.838 (0.822-4.112)	0.138		
Smoke (yes/no)	1.564 (0.671-3.647)	0.301		

**Table 2:** Univariate and Multivariate cox regression analysis-GEO cohort.

Variables	Univariate analysis		Multivariate analysis	
	HR (95%CI)	P value	HR (95%CI)	P value
Risk score (high/low)	1.8663 (1.1557-2.9832)	0.0091	1.6915 (1.053-2.717)	0.0297
Stage (III/I+II)	2.1895 (1.339-3.582)	0.0018	1.4969 (0.753-2.974)	0.2495
Grade (2 + 3/1)	1.0040 (0.56-1.8)	0.989		
pT (3 + 4/1+2)	0.9634 (0.5656-1.641)	0.891		
pN (1 + 2+3/0)	2.1594 (1.319-3.535)	0.0022	1.5383 (0.776-3.050)	0.2175
Gender (Male/Female)	0.8269 (0.4681-1.461)	0.513		
Age (≥65/65)	1.535 (0.941-2.503)	0.0861		
Smoke (yes/no)	1.1634 (0.7203-1.879)	0.536		

### The Different Immune Infiltration Between High- and Low-Risk Group

**Figure 7:**
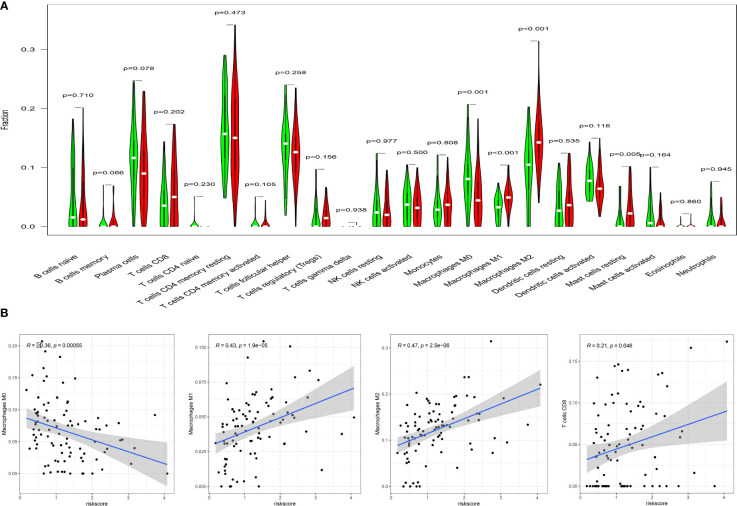
Correlation of TICs with risk score model. **(A)** Violin diagram for the levels of immune cells between high-risk group(red) and low-risk group (green). **(B)** There were significant correlations between risk score model and macrophage M0,M1,M2 and T cells CD8. Pearson test was used for the correlation test (p<0.05).

We estimated the difference of immune infiltration between high- and low-risk ESCC patients in 22 subpopulations of immune cells using the CIBERSORT algorithm. Levels of M1 macrophages (p <0.001) and M2 macrophages (p < 0.001) were markedly higher in the high-risk compared to the low-risk group. Additionally, the proportion of M2 macrophages were significantly higher than M1 macrophages (**Figure 7A**). In contrast, a high fraction of resting CD4 memory T cells, activated dendritic cells, and M0 macrophages mainly infiltrated low-risk ESCC patients. The correlation analysis further confirmed the risk score was moderately correlated with M1 macrophages (r = 0.43, p<0.0001) and M2 macrophages (r = 0.47, p <0.0001). Moreover, there were weak associations between risk score and CD8 T cells (r=0.21, p=0.046), and a negative correlation between risk score and M0 macrophages (r=-0.36, p<0.001, **Figure 7B**). These results indicate that differences in immune infiltration in high- and low-risk patients with ESCC might be used as a prognostic indicator and target for immunotherapy.

### Establishment and Validation of a Nomogram

**Figure 8:**
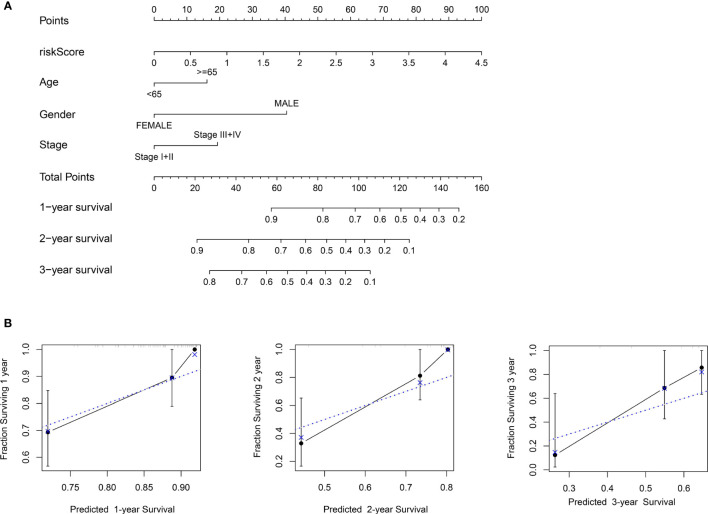
Construction of the nomogram. **(A)** Nomogram predicting 1-, 2-, and 3- year survival based on risk score and other clinical parameters. **(B)** The calibration curves of nomogram between predicted and actual 1-, 2- and 3-year OS in the training cohort. The blue dotted line represents perfect prediction of the nomogram.

To establish a more convenient and applicable clinical prognostic approach, we developed a nomogram based on our risk score and other clinical characteristics including age, gender and pathologic stage (**Figure 8A**). The concordance index of the nomogram was 0.734. The calibration plot for the possibility of 1-, 2- and 3-year survival showed good agreement between the prediction and actual observations (**Figure 8B**). These findings illustrate that the nomogram may be a more effective method to predict prognosis of ESCC patients for clinicians.

### Differences in Immune-Related Pathways Between High- and Low-Risk Groups

**Figure 9:**
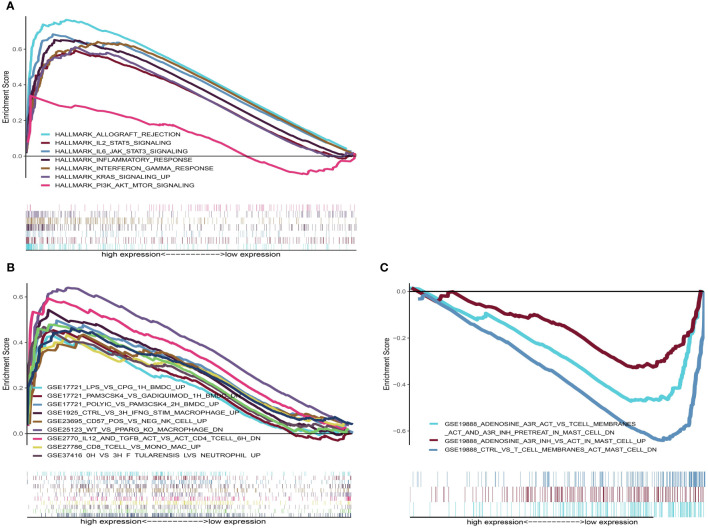
GSEA analysis for patients with high-risk and low-risk. **(A)** The enriched gene sets in HALLMARK collection in high-risk group. Each line representing one particular gene set with unique color. Only gene sets with NOM p < 0.05 and FDR q < 0.25 were considered significant. Only several leading gene sets were displayed in the plot. **(B)** The enriched gene sets in C7 collection (the immunologic gene sets) in high-risk group. **(C)** Enriched gene sets in low-risk group of C7 collection.

To help illustrate the underlying mechanism of how these genes impact patient outcomes, GSEA was utilized to evaluate different expression profiles among the two groups. Results showed that genes in the high-risk group were mainly enriched in several immune-related pathways, such as allograft rejection, IL2_STAT5_SIGNALING, IL6_JAK_STAT6_ SIGNALING, KRAS_ SIGNALING_UP and PI3K_AKT_MTOR_SIGNALING (**Figure 9A**). However, no significant gene sets were enriched in the low-risk group. For the C7 immunologic gene sets defined by MSigDB, multiple immune functional gene sets were enriched in high-risk group (**Figure 9B**) while only three gene sets were enriched in the low-risk group (**Figure 9C**). These results suggest that these genes might affect tumor immune status through these pathways.

### qRT-PCR Validation

**Figure 10:**
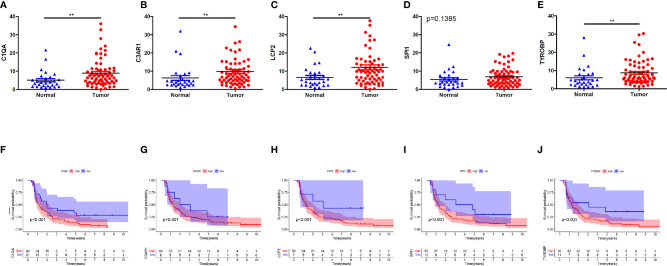
Five genes validation using qRT-PCR for ESCC cDNA microarray chip. **(A–E)** Expression levels of C1QA, C3AR1, LCP2, SPI1, TYROBP were up-regulated in tumor tissues. **(F–J)** The expression of C1QA, C3AR1, LCP2, SPI1, TYROBP were correlated with poor outcomes of ESCC patients. (p<0.001). **p < 0.01.

**Figure 11:**
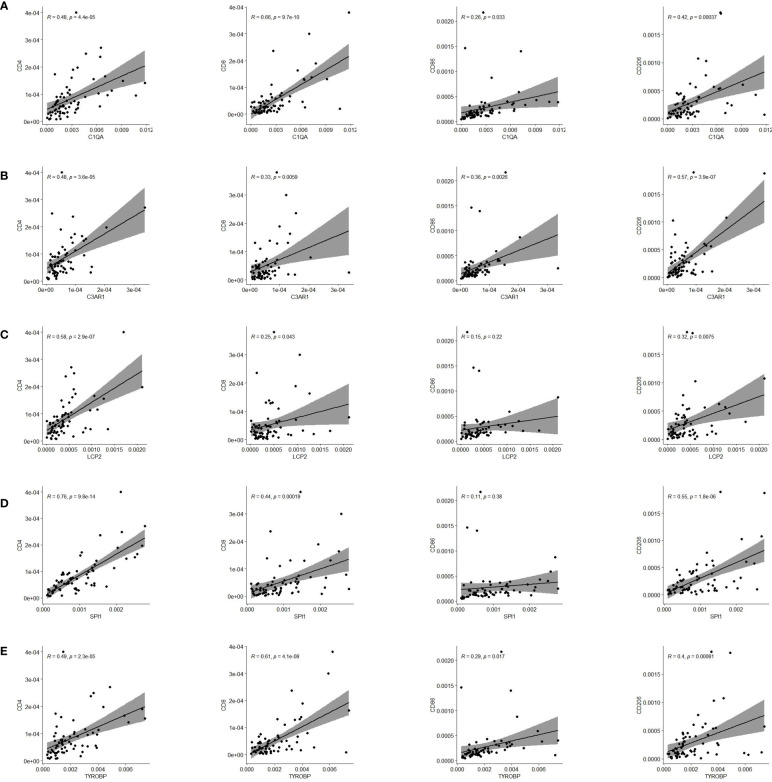
Pearson correlation of five genes expression and immune cells surface biomarkers. **(A)** C1QA is tightly related to CD4, CD8 and CD206. **(B)** C3AR1 is significantly correlated with CD4 and CD206. **(C)** LCP2 has close relationship with CD4 and CD206. **(D)** SPI1 is significantly linked with CD4 and CD206. **(E)** TYROBP is tightly related to CD4, CD8 and CD206.

Finally, we validated five rarely reported genes among these ten genes using an ESCC cDNA microarray by qRT-PCR. As demonstrated in **Figures 10A–E**, the relative expression level of C1QA, C3AR1, LCP2 and TYROBP in ESCC samples were significantly higher relative to normal samples (p < 0.05). The expression of SPI1 was also up-regulated although not statistically significant. The survival curve showed that they were all significantly associated with the overall survival of ESCC (log-rank p < 0.05; **Figures 10F–J**). In addition, based on the CIBERSORT results, we further examined the correlation between 5 genes and macrophage M1 and M2 surface markers. As depicted in **Figure 11**, compared with CD8 and CD86, they had stronger correlation with CD206 and CD4, which further verified they may be involved in the activity of M2 macrophages and play an immunosuppressive role in TME.

## Discussion

Esophageal squamous-cell carcinoma (ESCC) is the most common type of esophageal cancer and approximately half of the world’s 500,000 new cases occur in China each year (5). Despite advancements in the treatment of ESCC, it continues to be a major challenge for public health worldwide (25). The role of immunotherapy in esophageal cancer is still poorly defined, largely due to high heterogeneity of tumor cells and the microenvironment (26–28). Previous studies have demonstrated that the TME has an important role in tumor progression and prognosis (29, 30).Thus, it is critical to unravel the immune infiltration of ESCC and identify potential predictive markers. In the present study, we identified 133 DEGs related to the TME, and 10 candidate genes were selected according to the intersection of PPI network and univariate cox analysis. Moreover, we constructed a 10-gene signature correlated with poor overall survival of ESCC patients. The immune infiltration demonstrated the signature had a close relationship with M2 macrophages. Finally, we validated five of the ten genes (C1QA, C3AR1, LCP2, SPI1 and TYROBP) as independently associated with poor survival and tightly related with M2 macrophage surface biomarkers, which may provide new therapeutic avenue for ESCC.

In our research, the ESTIMATE algorithm was utilized to estimate the immune/stromal components of the TME. Our results showed that the immune/stromal scores were both significantly associated with the OS of patients, indicating that TME composition affects the outcomes of ESCC patients. Furthermore, through differential analysis, 133 up-regulated immune and stromal genes were identified. Following functional enrichment analysis of DEGs, they were mainly involved in several immune activities such as the Natural killer cell mediated cytotoxicity, Toll−like receptor signaling pathway, Th17 cell differentiation and so on. These results may give further clues about ESCC etiology and progression. For instance, it has been shown that lipopolysaccharide (LPS)-induced TLR4 signaling promotes cancer cell proliferation and contributes to cancer development and progression in ESCC (31). Next, we constructed a PPI network and univariate cox analysis based on these genes, 10 hub genes were extracted. The above genes were verified using data from the GEO database, and the expression of 9 genes (excluding CSF1R) were significantly up-regulated in the tumor tissues compared with the paired normal tissues. Moreover, they were lowly-expressed in esophageal cancer cell lines compared with classic epithelial markers, and significantly positively associated with ImmuneScore while negatively linked with TumorPurity, suggesting that these genes may be specifically expressed in immune cells. Next, we established a 10-gene signature based on the multivariate cox analysis. The AUC for the 10-gene signature for predicting the 1-, 2-, and 3-year survival were 0.672, 0.854 and 0.81, respectively. The signature was further validated in the GEO dataset. The results suggested that the ten-gene signature was an independent prognostic factor and had a good performance for survival prediction.

In addition, a nomogram was established including the 10-gene signature and age, gender, amd pathologic stage to more accurately predict survival probability. The calibration plot showed good agreement between the prediction by risk score and actual observation. GSEA results showed that several immune-related pathways were enriched in the high-risk group, indicating these ten genes may influence patients’ prognosis through these immune response processes. For instance, IL-2 signaling is an essential and multi-functional regulator of many immune cell populations, including effector and regulatory CD4+ T cell subsets *via* activation of Signal Transducer and Activator of Transcription 5 (STAT5). It’s also required for differentiation and functional maturation in early Treg stages. This pathway has long been the target of therapeutic strategies to treat diseases ranging from cancer to autoimmunity (32). JAK-STAT signaling is activated by a number of cytokines including IL-6, TNF-α, and IFN-γ and has been found to be involved in regulation of cell proliferation, differentiation and apoptosis (33). A previous study reported that it is associated with the progression of colorectal cancer (34). KRAS is one of the most frequently mutated oncogenes in cancer, being a potent initiator of tumorigenesis, and a predictive target of response to therapy (35). Importantly, Lastwika et al. showed that the activation of KRAS-downstream pathway PI3K/AKT/mTOR is tightly linked with the regulation of PD-L1 expression both *in vitro* and *in vivo* for human LACs and squamous cell carcinomas, indicating that KRAS may cause immune escape by AKT/mTOR pathway *via* PD-L1 (36).Our findings demonstrated that the 10-gene signature have a close relationship with macrophage M2, which indicates that macrophage M2 might be involved in progression and poor survival outcomes in ESCC. Tumor-associated macrophages (TAMs) have been reported to play an important role in modulating the tumor-microenvironment to an immune suppressive mode, promoting tumor growth, angiogenesis, invasion, and metastasis as well as resistance to therapy (37). Colony-stimulating factor 1 receptor (CSF1R), included in our gene signature, a class III receptor tyrosine kinase, is confirmed to promote TAM transition to a pro-tumorigenic M2-phenotype through binding macrophage colony stimulating factor 1 (MCSF) cytokines (38). As the presence of CSF1R+ macrophages correlates with poor survival in various tumor types (39), a variety of small molecules and monoclonal antibodies (mAbs) directed at CSF1R are in clinical development, which represents an attractive strategy in tumor therapy (40).

We further carried out some experiments to validate five rarely reported genes, which could be used as potential biomarkers for future treatment of ESCC. According to the survival analysis from a cDNA microarray, five genes were up-regulated in tumor samples and predict poor prognosis in ESCC patients. TYROBP, also known as KARAP/DAP12 (killer cell activating receptor‐associated protein/DNAX activating protein of 12 kDa), has been found to be linked with the poor prognosis and skeletal metastasis of breast cancer (41). It was also proved that a DAP12‐dependent NK cell receptor NKG2D is involved in antitumoral activity and regulates NK cell function according to experiments with TYROBP knockout mice (42). Consistently, our findings revealed that TYROBP and related genes are mainly enriched in immune-related activities, such as NK cell‐mediated cytotoxicity. In the present study, TYROBP had a remarkable positive correlation with M2 macrophages in ESCC. This is consistent with a previous studies indicating that TYROBP is positively linked with M2 macrophages, and may play an important role in immunosuppression and differentiation of TAMs into M2 macrophages in the tumor microenvironment (43). C1q, the first recognition subcomponent of the complement classical pathway, includes three chains (C1qA, C1qB, and C1qC) and has been proved to highly expressed in multiple tumors, including prostate (44), and mesothelioma (45) as well as gliomas (46).In our study, the expression of C1QA were significantly correlated with ESCC patients’ survival and upregulated in tumor samples. Our results suggested that C1QA may participate in the regulation of CD8+ T cells, Tregs and M2 macrophages in the immune infiltration of ESCC. Consistently, C1QA and C1QB were confirmed to be drivers of alternatively activated macrophage polarization in a LPS-induced inflammation model (47). Samantha et al. (48) reported that macrophages express high levels of C1QA and C1QB in both primary tumor and metastases. C3AR1 as a receptor of the complement effector C3a, is important in mediating the downstream signal transduction of the complement activation. A previous study had demonstrated that a high concentration of C3a in the serum of esophageal cancer patients was associated with a poor prognosis (49). Meanwhile, there were moderate associations between C3AR1 expression and M1 macrophages, M2 macrophages, CD8 T-cells, and Tregs. A previous indicated that Toll-like receptor(TLR)-initiated DC autocrine C3AR1 signaling causes expansion of effector T cells and instability of regulatory T cells (50). It has been found that C3AR1 can promote the polarization of M2 macrophages and T cell exhaustion, leading to the immune escape of STAD (51). Lawal et al. reported that C3AR1 was associated with tumor immune evasion, prognosis, and immunotherapy in melanoma, colorectal, brain, breast, stomach, and renal cancer (52). We speculated that C3AR1 may play a similar role in tumor immunity and promoting the development of ESCC. SPI1(PU.1) is a TF crucial for normal T-cell maturation. It is reported that SPI1 is a potent inducer of granulocytic/monocytic differentiation and is often expressed at a low level in AML (53). Gongwei et al. confirmed that overexpression of SPI1 effectively suppresses the growth of MYC-deregulated B-cell lymphomas (54). Alterations in SPI1 lead to oncogenic subversion by cellular proliferation and differentiation arrest in Waldenström macroglobulinemia (55). SPI1 has previously been demonstrated to be associated with the development of different types of immune lineage cells, consisting T-cells, B-cells, monocytes and dendritic cells (56–58). There are a few reports about its role in solid tumors. For instance, Gao et al. proved that SPI1-induced upregulation of SNHG6 promoted the cellular processes in NSCLC *via* miR-485-3p/VPS45 axis (59). In our present study, the expression of SPI1 were significantly up-regulated in tumor tissues and correlated with M2 macrophages, CD8 T cells, Tregs, and predicts poor survival of ESCC patients, suggesting that SPI1 may be a potential biomarker of ESCC and play an equally crucial role in solid tumors. LCP2 is known to participate in T cell progression and it was previously reported that a splice variant of LCP2 resulted in severe immune dysregulation (60). High expression levels of LCP2 contributed to poor outcomes in ESCC patients in our study. Moreover, a previous study confirmed that lncRNA ITGB2-AS1 can promote the migration and invasion of breast cancer cells by up-regulating ITGB2 (61). Zhang et al. uncovered the higher ITGB2 expression in CAFs promote tumor proliferation in OSCC by activating the PI3K/AKT/mTOR pathways and NADH oxidation in the mitochondrial oxidative phosphorylation system (62), and ITGB2 was found to be involved in the proliferation, migration, and invasion of CRC cells (63).

In this study, using the ESTIMATE algorithm, we developed a 10-gene signature to predict the prognosis of ESCC patients. Our TME-related ten-gene prognostic signature was confirmed to have good predictive performance and to represent an independent prognostic factor. We also validated five genes (C1QA, C3AR1, LCP2, TYROBP, SPI1) using cDNA microarray, which could be potential biomarkers for future treatment of ESCC. To our knowledge, this is the first time report of an M2 macrophage-related prognostic gene signature in ESCC. However, there are several limitations. To be useful, this prognostic signature needs to be further validated in some large cohorts and multicenter clinical trials in the future. Additionally, further experiments are required to clarify the potential mechanism and the specific roles of these TME-related genes in the development, migration, and invasion of ESCC.

## Conclusion

In summary, we established and validated a novel gene signature that is based on ten immune-related genes to predict the OS of ESCC. This signature is significantly correlated with M2 macrophages in the tumor microenvironment. Notably, C1QA, C3AR1, LCP2, TYROBP and SPI1 were further validated as up-regulated in tumors and independently predict poor outcomes in ESCC. Hence, they may be underlying therapeutic targets for ESCC and are expected to be further applied in future clinical practice.

## Data Availability Statement

The datasets presented in this study can be found in online repositories. The names of the repository/repositories and accession number(s) can be found in the article/**Supplementary Material**.

## Author Contributions

JY and LD contributed equally to this work. JY, LD, YG, and BH designed the research; XH, XF, JL, RY, ZX, and HL performed the experiments; JY, LD, and GA analyzed the data. All authors wrote the paper; YG and BH revised the paper. All authors contributed to the article and approved the submitted version.

## Funding

This work was supported by National Natural Science Foundation of China (82003057) to Jiannan Yao), and National Natural Science Foundation of China (81802738) to YG.

## Conflict of Interest

The authors declare that the research was conducted in the absence of any commercial or financial relationships that could be construed as a potential conflict of interest.

## Publisher’s Note

All claims expressed in this article are solely those of the authors and do not necessarily represent those of their affiliated organizations, or those of the publisher, the editors and the reviewers. Any product that may be evaluated in this article, or claim that may be made by its manufacturer, is not guaranteed or endorsed by the publisher.
