# Supplementary figures and images for: Development and Validation of a Prognostic Gene Signature Correlated With M2 Macrophage Infiltration in Esophageal Squamous Cell Carcinoma
Source: Front Oncol. 2021 Dec 3;11:769727. doi: 10.3389/fonc.2021.769727 (PMC8677679; doi:10.3389/fonc.2021.769727)

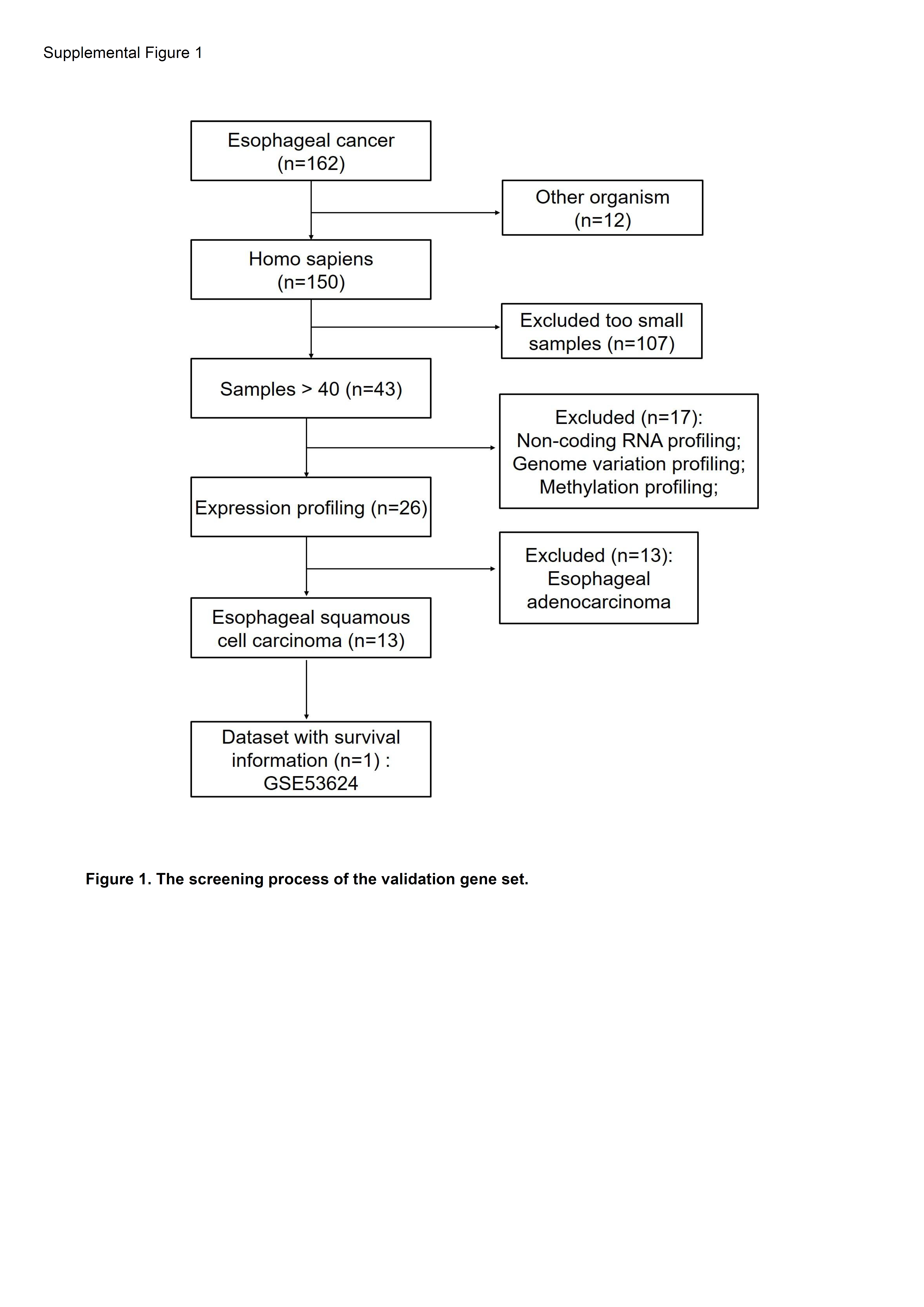

Supplement: Supplementary file 1 [file Image_1.tif]

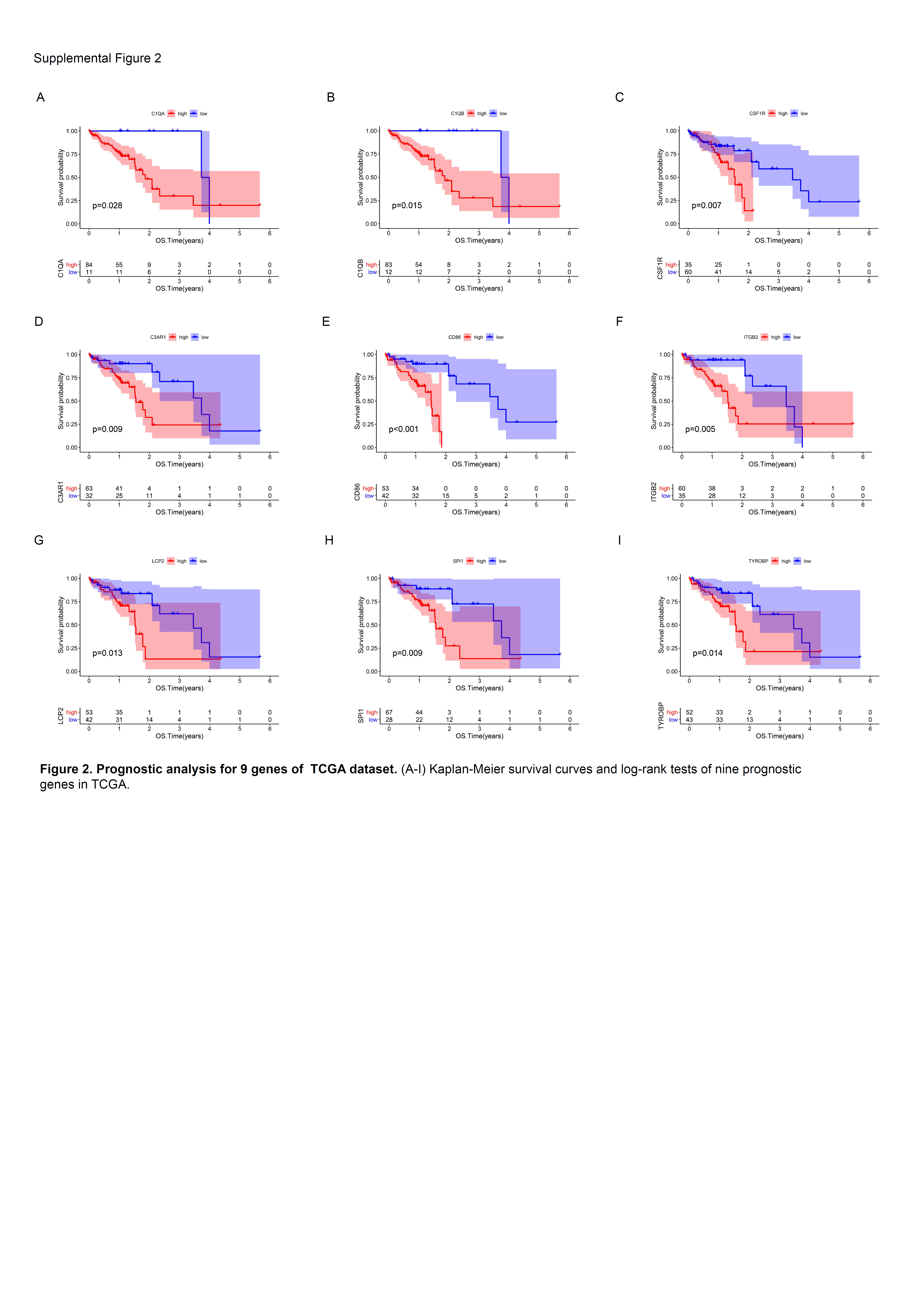

Supplement: Supplementary file 2 [file Image_2.tif]

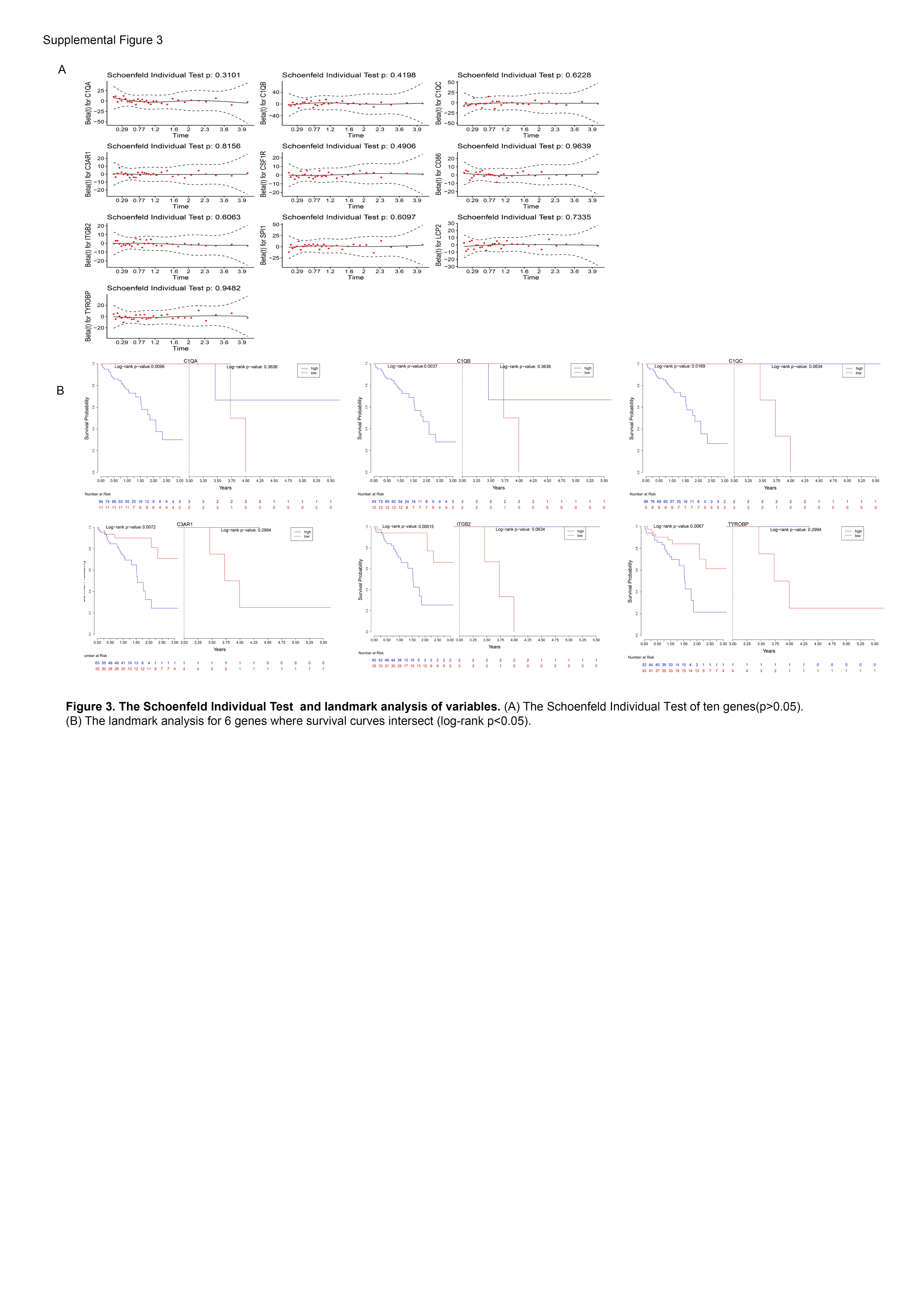

Supplement: Supplementary file 3 [file Image_3.tif]

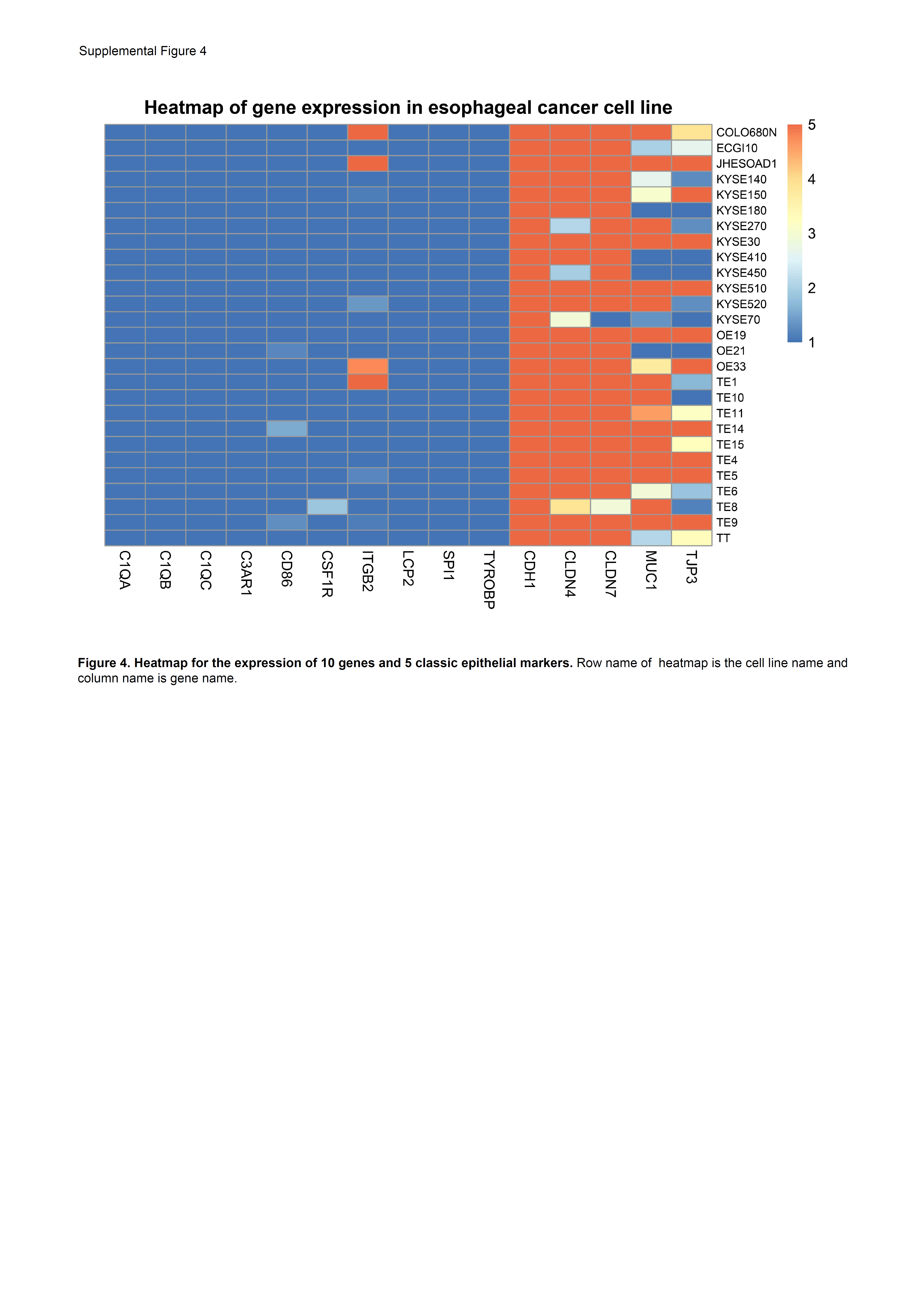

Supplement: Supplementary file 4 [file Image_4.tif]

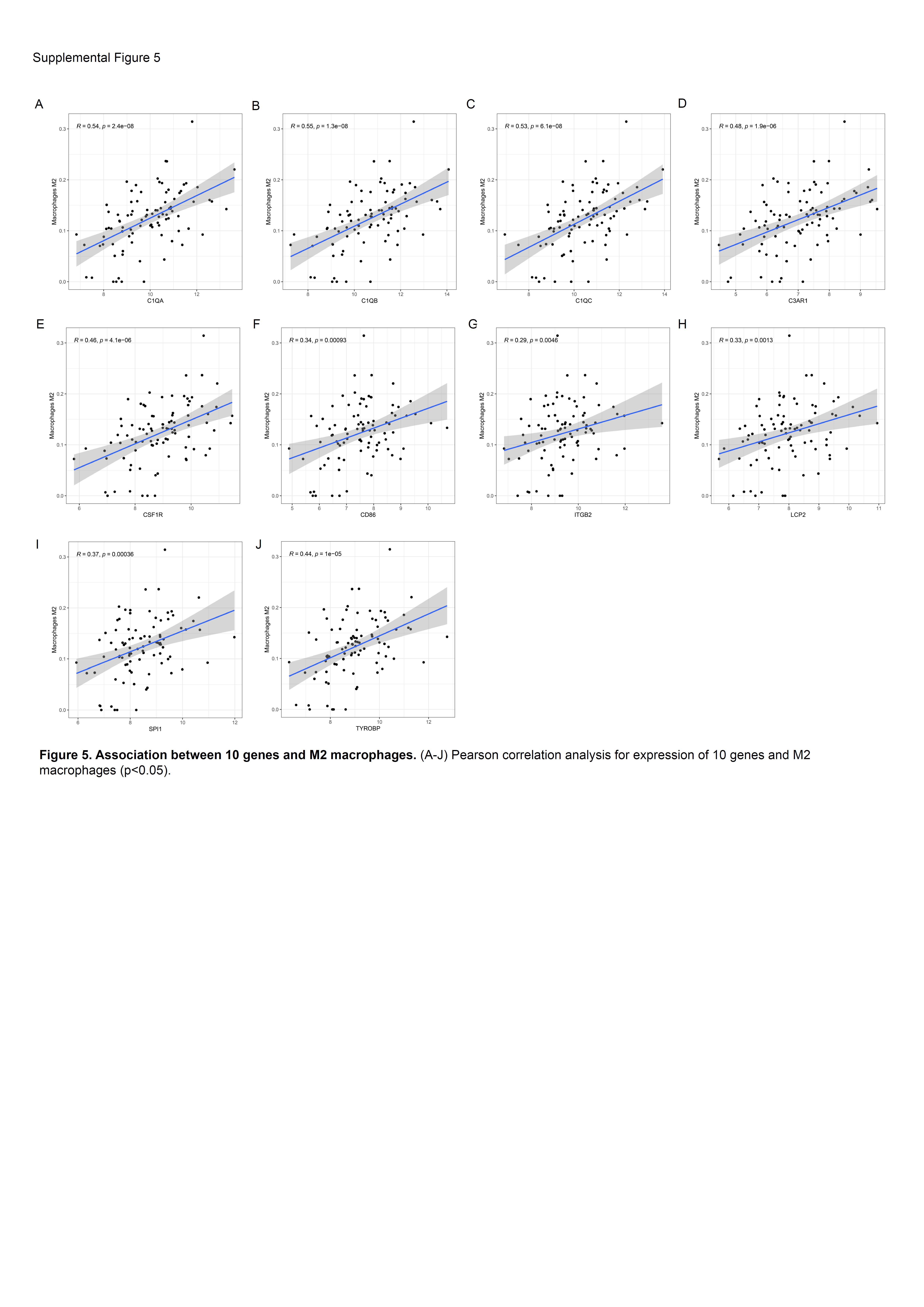

Supplement: Supplementary file 5 [file Image_5.tif]

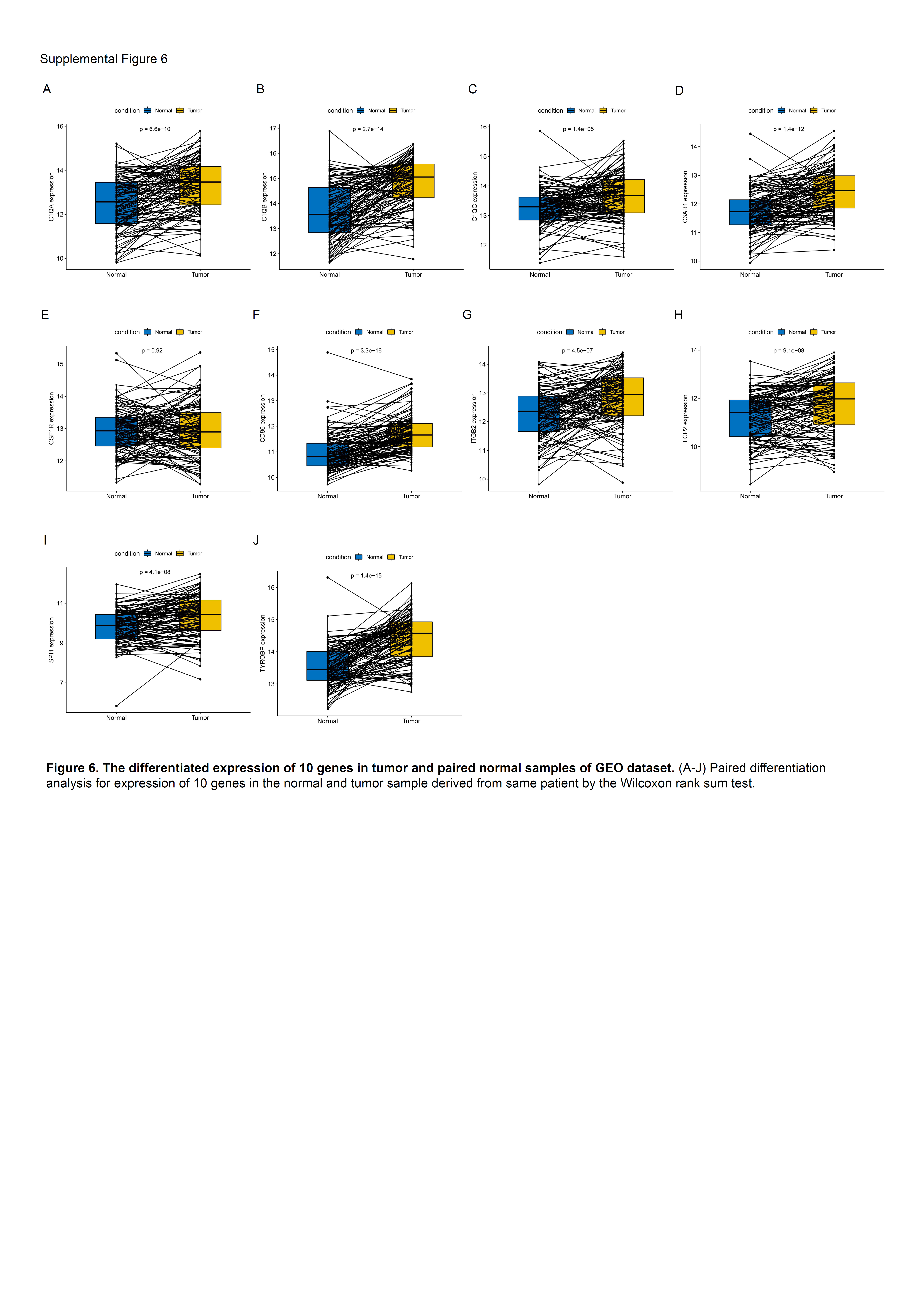

Supplement: Supplementary file 6 [file Image_6.tif]
