# Supplementary material for: Development and Validation of a Prognostic Gene Signature Correlated With M2 Macrophage Infiltration in Esophageal Squamous Cell Carcinoma
Source: Front Oncol. 2021 Dec 3;11:769727. doi: 10.3389/fonc.2021.769727 (PMC8677679; doi:10.3389/fonc.2021.769727)
Supplement: Supplementary file 7 [file Table_1.docx]

| **Supplemental Table 1. Clinical characteristics of ESCC patients in TCGA** | |
| --- | --- |
| characteristics | N |
| All patients | 95 |
| Gender  Male  Female | 14  81 |
| Age  <65  >=65 | 71  24 |
| Histologic Grade  Low (G1+G2)  High G3  NA | 64  21  10 |
| Tumor  Low (T1+T2)  High (T3+T4)  NA | 39  54  2 |
| Node  Positive  Negative  NA | 38  54  3 |
| Metastasis  Positive  Negative  NA | 4  83  8 |
| Pathologic Stage  Early (I+II)  Advanced (III+IV)  NA | 62  31  6 |
| Smoke  Yes  No  NA | 59  32  4 |

| **Supplemental Table 2. Specific primers used in real-time PCR analysis** | | |
| --- | --- | --- |
| Gene | Primer | Sequence (5*'*→3*'*) |
| C1QA | FW | TCTGCACTGTACCCGGCTA |
|  | RV | CCCTGGTAAATGTGACCCTTTT |
| C3AR1 | FW | CCCTACGGCAGGTTCCTATG |
|  | RV | GACAGCGATCCAGGCTAATGG |
| LCP2 | FW | GGAATGTGCCCTTTCGCTCA |
|  | RV | TCCTCCTCTCTTCGTTCTTGTT |
| SPI1 | FW | GTGCCCTATGACACGGATCTA |
|  | RV | AGTCCCAGTAATGGTCGCTAT |
| TYROBP | FW | ACTGAGACCGAGTCGCCTTAT |
|  | RV | ATACGGCCTCTGTGTGTTGAG |
| CD4 | FW | TGCCTCAGTATGCTGGCTCT |
|  | RV | GAGACCTTTGCCTCCTTGTTC |
| CD8 | FW | ATGGCCTTACCAGTGACCG |
|  | RV | AGGTTCCAGGTCCGATCCAG |
| CD86 | FW | CTGCTCATCTATACACGGTTACC |
|  | RV | GGAAACGTCGTACAGTTCTGTG |
| CD206 | FW | CTACAAGGGATCGGGTTTATGGA |
|  | RV | TTGGCATTGCCTAGTAGCGTA |
